# Early, midday, and late time-restricted eating impact on anthropometry and cardiometabolic health: a network meta-analysis of RCTs

**DOI:** 10.3389/fnut.2026.1887087

**Published:** 2026-08-06

**Authors:** Mohammed Hamsho, Wijdan Shkorfu, Merve Terzi, Yazan Ranneh, Krista A. Varady, Abdulmannan Fadel

**Affiliations:** 1 Department of Nutrition and Dietetics, Faculty of Health Sciences, Istanbul Yeni Yuzyil University, Istanbul, Türkiye; 2 Department of Nutrition and Dietetics, Faculty of Health Sciences, Bahçeşehir University, Istanbul, Türkiye; 3 Department of Nutrition and Dietetics, College of Pharmacy, Al-Ain University, Abu Dhabi, United Arab Emirates; 4 Department of Kinesiology and Nutrition, University of Illinois Chicago, Chicago, IL, United States; 5 Department of Nutrition and Health, College of Medicine and Health Sciences, United Arab Emirates University, Al Ain, United Arab Emirates

**Keywords:** calorie restriction, circadian rhythm, early time-restricted eating, intermittent fasting, late time-restricted eating, midday time-restricted eating, network meta-analysis, time-restricted eating

## Abstract

**Objective:**

This network meta-analysis (NMA) evaluates the physiological effects of early time-restricted eating (eTRE), midday TRE (mTRE), and late TRE (lTRE), with and without exercise (EX), calorie restriction (CR), and control on anthropometric and metabolic indices in adults with cardiometabolic risk factors.

**Methods:**

A comprehensive literature search was conducted in PubMed, Web of Science, Scopus, and Embase up to April 24, 2025. A Bayesian NMA was performed. Treatment effects were expressed as mean differences (MD) with 95% credible intervals (CrI). Intervention rankings were assessed using the surface under the cumulative ranking curve (SUCRA). Certainty of evidence was evaluated using the GRADE approach.

**Results:**

Thirty-nine randomized controlled trials including 3,236 participants were analyzed. Most interventions, except mTRE + EX, reduced anthropometric measures compared with control. eTRE reduced fasting blood glucose (MD −3.64 mg/dL, 95% CrI −5.93 to −1.36) and HOMA-IR (MD −0.42, 95% CrI −0.83 to −0.03), compared with control group. eTRE + EX reduced HOMA-IR (MD −1.33, 95% CrI −2.58 to −0.08). Across outcomes, eTRE + EX and eTRE consistently ranked highest according to SUCRA. Certainty of evidence ranged from very low to moderate for most anthropometric and metabolic outcomes.

**Conclusion:**

Although clear differences between TRE patterns and CR were limited, consistent SUCRA rankings favored eTRE, particularly when combined with exercise, which may suggest a potential role of meal timing in metabolic regulation. However, these rankings should be interpreted cautiously, as they were based on a limited number of trials, and the certainty of evidence ranged from very low to moderate for most outcomes.

**Systematic review registration:**

https://www.crd.york.ac.uk/PROSPERO/view/CRD420251018349.

## Graphical abstract

**Figure:**
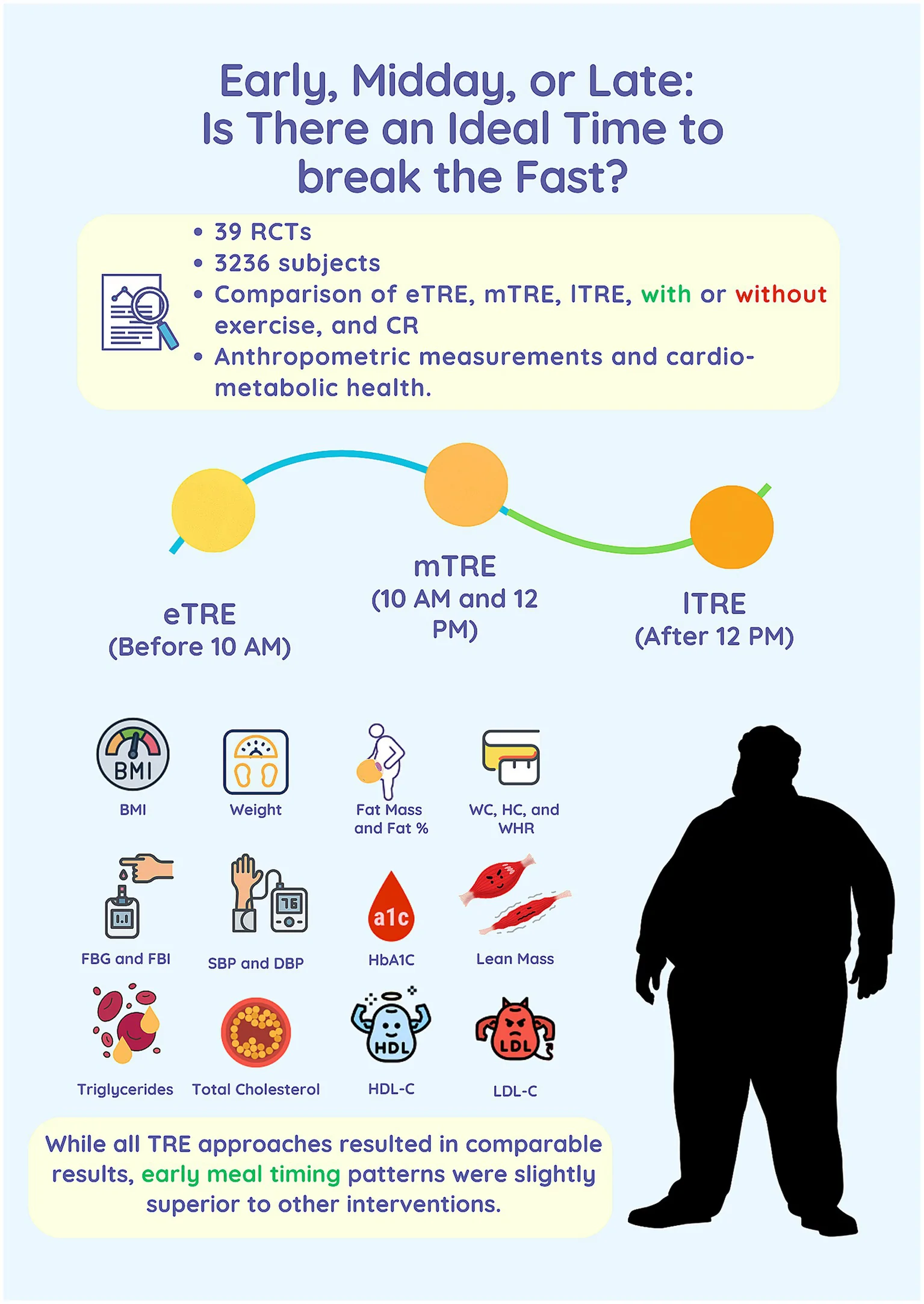

## 1 Introduction

Time-restricted eating (TRE) is a pattern of intermittent fasting that focuses more on WHEN you should eat, instead of restricting how much you should eat (1). TRE generally consists of consuming all of your daily energy intake within a certain time period each day, ranging anywhere from 6–10 h, followed by fasting for the remaining hours of the day (1). While TRE may sound quite simplistic, studies have shown that the timing of eating and fasting have direct effects on human biomarkers (2). TRE is thought to play an important role by syncing feeding cycles to the body’s endogenous circadian rhythm (2, 3). The circadian rhythm controls various biological functions that occur throughout the 24-h day cycle, many of which vary depending on the time of day, including insulin release, glucose metabolism, and energy expenditure (4, 5).

According to the theory behind TRE, our bodies are able to metabolize nutrients most efficiently during the daytime hours (2, 6, 7). Eating later in the day or night can cause this eating pattern to become de-synced from your circadian rhythm. Nowadays, many eat inconsistently throughout the day and night. Eating late at night tends to cause individuals to consume more calories over a longer period of time which could lead to over consumption of energy and further de-sync circadian rhythms of peripheral clocks found in other metabolically active organs leading to disruption of lipid metabolism, glucose regulation, and insulin regulation (6). This can cause negative metabolic effects and raise risk of metabolic syndrome, diabetes, and CVD (2, 3).

Coincidently, breakfast skipping has been linked to increases in daily energy intake later in the day in observational studies (8, 9). TRE is a purposeful eating behavior that clusters energy intake into defined eating windows earlier in the day aligning with the body’s circadian physiology. Recently, a meta-analysis demonstrated that cluster feeding earlier in the day is associated with lower BW and BMI than feeding later in the day (10).

To date, several clinical trials have explored differential impacts of TRE timing using primarily two models, early TRE (eTRE) and late or midday TRE (lTRE/mTRE) where all meals are restricted to the early morning to midday and midday to evening, respectively (11–16).

For the current NMA, we expanded upon previous work by further stratifying dietary timing into three TRE models (early, midday, and late TRE) to assess the impact of meal timing on clinical outcomes. While previous evidence indicates differential effects of early vs. late-phase eating models on insulin sensitivity, blood pressure, and oxidative stress in favor of early-phase models under isocaloric conditions without weight loss (17), these benefits may be potentiated with the inclusion of CR (18–20). Moreover, as multiple studies have evaluated the effects of exercise paired with differing TRE models (21–25), and exercise may impact TRE-mediated effects, we included exercise as a secondary modifier while also independently evaluating meal timing. Thus, our analysis allowed for simultaneous consideration of eating and exercise timing.

Therefore, we performed a systematic review and network meta-analysis (NMA) of randomized controlled trials (RCTs) that investigated the effects of time-restricted eating (TRE) at different times of the day [early (eTRE), midday (mTRE), and late (lTRE)] with or without exercise and calorie restriction (CR) on anthropometric and cardiometabolic outcomes in adults at risk of CVDs.

## 2 Methods

The study protocol was registered in PROSPERO with ID: CRD420251018349. Interventions definitions were clearly explained in Supplementary Table S1.

### 2.1 Search strategy

Online databases (PubMed, Web of Science, Scopus, Embase) were searched up until 24 April 2025 by two authors independently (AF and YR). We conducted a systematic search of the literature to identify randomized controlled trials investigating the effect of TRE interventions differing in timing and/or duration on anthropometric measurements, metabolic profile and blood pressure. The search strategy was reported in (Supplementary Table S2). In addition, the reference lists of all eligible studies were manually screened to identify any potentially relevant studies that may not have been retrieved through the electronic database search.

### 2.2 Eligibility criteria

Studies were considered eligible if they reported on adult patients with cardiometabolic risk factor(s), including overweight patients (BMI 25.0–29.9 kg/m^2^), obese patients (BMI ≥ 30.0 kg/m^2^), patients with normal BMI (18.5–24.9 kg/m^2^) but hidden obesity (body fat % ≥ 30%), and patients with cardiometabolic-related chronic diseases including metabolic syndrome, non-alcoholic fatty liver disease (NAFLD), and type 2 diabetes (T2D) who completed interventions using one of our prespecified forms of TRE (early TRE (eTRE) where first caloric intake occurs before 10: 00 a.m., mid-day TRE (mTRE) where first caloric intake occurs between 10:00 a.m.–12:00 p.m., or late TRE (lTRE) where first caloric intake occurs after 12:00 p.m. noon) or the same TRE schedules with structured exercise (eTRE + EX, mTRE + EX, and lTRE + EX). All TRE interventions needed to include a daily fasting duration of ≥ 14 h, must be implemented on ≥ 6 days per week, and required ≥ 1 month duration. Control groups had to consume diets without prescribed eating or fasting windows (e.g., ad libitum intake, usual care, no intervention), calorie restriction without time restriction, or undergo alternative prespecified TRE schedules (e.g., eTRE, mTRE, lTRE, eTRE + EX, mTRE + EX, and lTRE + EX). We considered anthropometric measures, measures of glucose metabolism, measures of lipid metabolism, and blood pressure outcomes. Lastly, studies needed to be randomized controlled trials published in English with no date restrictions.

Studies were excluded if they focused on pregnant women, professional athletes or other highly physically active populations (defined as studies where participants were engaged in structured exercise training ≥ 6 months before study enrollment), night-shift or rotating-shift workers, exclusively enrolled participants with normal BMI/body fat percentage who were free of chronic diseases, included eating windows > 10 h/day, included fasting durations < 14 h/day, utilized self-selected/non-prescribed eating windows, or had intervention periods focused primarily on weight maintenance (e.g., included an initial intervention phase with a subsequent weight-maintenance phase). Studies with insufficient data to perform statistical analysis (e.g., studies that provided post-intervention data only) were also excluded (Table 1).

**Table 1:** PICO framework defining the eligibility criteria used for study selection.

PICOS framework	Inclusion criteria	Exclusion criteria
Population (P)	A) Adult participants with cardiometabolic risk factor(s), including individuals with overweight (BMI 25.0–29.9 kg/m^2^), obesity (BMI ≥ 30.0 kg/m^2^), or normal BMI (18.5–24.9 kg/m^2^) with hidden obesity (body fat ≥ 30%), as well as those with cardiometabolic-related chronic conditions such as metabolic syndrome, non-alcoholic fatty liver disease, and type 2 diabetes.	B) Pregnant women C) Performing exercise for at least 6 months prior to study (e.g., professional athletes and reactionary active subjects) D) Night shift workers E) Studies exclusively include participants with normal BMI and normal body fat percentage who were free from chronic diseases.
Intervention (I)	A) Time-restricted eating (TRE) implemented as one of the prespecified forms 1) Early TRE (eTRE): first caloric intake before 10:00 a.m.; 2) Md-day TRE (mTRE): first caloric intake between 10:00 a.m. and 12:00 p.m.; 3) Late TRE (lTRE): first caloric intake after 12:00 p.m. 4) Early TRE (eTRE): first caloric intake before 10:00 a.m. combined with exercise (eTRE + EX); 5) Midday TRE (mTRE): first caloric intake between 10:00 a.m. and 12:00 p.m. combined with exercise (mTRE + EX), 6) Late TRE (lTRE): first caloric intake after 12:00 p.m. combined with exercise (lTRE + EX), all defined by a daily fasting duration of ≥14 h implemented on ≥ 6 days per week, with or without exercise.	A) Eating window > 10 h or fasting window <14 h a day. B) Self-selected eating windows. C) Follow up periods aiming for weight maintenance (e.g., 3 months intervention and 3 months follow up weight maintenance)
Comparison (C)	A) Control conditions without prescribed eating or fasting windows (e.g., ad libitum intake, usual care, or no intervention), calorie restriction without time restriction, or alternative TRE schedules (eTRE, mTRE, lTRE, eTRE + EX, mTRE + EX, or lTRE + EX).	A) Prescription of structured exercise to the control group.
Outcome (O)	A) Anthropometric measurements, glucose metabolism, lipid metabolism, and blood pressure.	Not applicable
Study setting (S)	A) Randomized controlled trials (RCTs) B) Minimum duration of 1 month	A) Insufficient data to perform statistical analysis (e.g., only post intervention data availability).
Language	English language published articles	
Date	No limits	

eTRE = early time-restricted eating, mTRE = midday time-restricted eating, lTRE = late time-restricted eating, eTRE + EX = early time-restricted eating + exercise, mTRE + EX = midday time-restricted eating + exercise.

### 2.3 Outcomes

A total of 18 outcomes were assessed: 1) BW (kg), 2) BMI (kg/m^2^), 3) fat %, 4) fat mass (kg), 5) lean body mass (LBM) (kg), 6) waist circumference (WC) (cm), 7) hip circumference (HC) (cm), 8) waist-to-hip ratio (WHR), 9) fasting blood glucose (FBG) (mg/dl), 10) fasting blood insulin (FBI) (mIU/L), 11) HOMA-IR, 12) hemoglobin A1c (HbA1c) (%), 13) triglycerides (TG) (mmol/l), 14) total cholesterol (TC) (mg/dl), 15) LDL-C (mg/dl), 16) HDL-C (mg/dl), 17) systolic blood pressure (SBP) (mmHg), and 18) diastolic blood pressure (DBP) (mmHg). Network meta-analysis was performed for each outcome.

### 2.4 Study selection

Study selection was conducted independently and in duplicate by two reviewers (MH and WS). The titles and abstracts of all identified records were screened to assess eligibility in stage 1, and the full texts of potentially eligible articles were obtained and assessed against inclusion and exclusion criteria in stage 2. All discrepancies were resolved through discussion or when needed, in consultation with a third senior investigator (YR). The study selection process was summarized in a PRISMA flow diagram, and reasons for exclusion were noted at each stage.

### 2.5 Data extraction

Following selection of studies based on the inclusion and exclusion, two reviewers (WS and MT) independently extracted the following information from each included study: study characteristics (first author, location, study design), participant characteristics (sample size, age, BMI, health status) and intervention characteristics (intervention(s), level of CR, control, intervention duration). These data are presented in Table 2.

**Table 2:** Characteristics of the randomized controlled trials included in the network meta-analysis.

Study	Study Llocation	Sample size (*n*)	Age (range) or (mean)	Sex	BMI	Health status	Study type	Interventions					Intervention duration
								Arm 1	Arm 2	Arm 3	Arm 4	Arm 5	
1) He et al. (11)	China	*n* = 169	18–65 y.o	F and M	29.3 kg/m^2^	MetS	RCT-parallel	LCD	8-h eTRE (ad libitum)	8-h lTRE (ad libitum)			3 months
2) Miladi et al. (50)	Tunisia	*n* = 75	> 18 y.o	F	> 25 kg/m^2^	Overweight or obese F	RCT-parallel	eTRE (1200–1,500 kcal) + EX (aerobic & resistance)	mTRE (1200–1,500 kacL) + EX (aerobic & resistance)	mTRE (1200–1,500 kcal)	Control (ad libitum)	/	12 weeks
3) Peter et al. (13)	Germany	*n* = 31	62 y.o (53–65 y.o)	F	30.5 kg/m2 (25–35 kg/m^2^)	Overweight or obese F	RCT-crossover	eTRE (ad libitum)	lTRE (ad libitum)	/	/	/	4-week
4) Xie et al. (13)	China	*n* = 90	18–64 y.o	F	17.5–30 kg/m^2^	Healthy without obesity	RCT-parallel	eTRE (ad libitum)	mTRE (ad libitum)	Control (ad libitum)	/	/	5-week
5) Queiroz et al. (19)	Brazil	*n* = 48	20–40 y.o	F and M	25–34.9 kg/m^2^	Overweight and obese adults	RCT-parallel	eTRE + CR by 25%	mTRE+ CR by 25%	CR only (−25%)	/	/	8-week
6) Zhang et al. (12)	China	*n* = 63	19–29 y.o	F and M	> 24 kg/m^2^	Overweight and obese young adults	RCT-parallel	6-h eTRE (ad libitum)	6-h mTRE (ad libitum)	Control (ad libitum)	/	/	8-week
7) Deng et al. (38)	China	*n* = 40	25–65 y.o	F and M	> 23 kg/m^2^ or <23 kg/m2 + MetS component	NAFLD	RCT-parallel	eTRE (ad libitum)	mTRE (ad libitum)	/	/	/	8-week
8) Dote-Montero et al. (15)	Spain	*n* = 197	30–60 y.o	F and M	25 < BMI < 40 kg/m^2^	Overweight and obese	RCT-parallel	eTRE (ad libitum)	lTRE (ad libitum)	Control	/	/	12-week
9) Liu et al. (10)	China	*n* = 139	18–75 y.o	F and M	28–45 kg/m^2^	Obese	RCT-parallel	eTRE + CR (1200–1,500 kcal for F, 1500–1800 kcal for M)	CR (1200–1,500 kcal for F, 1500–1800 kcal for M)	/	/	/	12 months
10) Liu et al. (21)	China	*n* = 80	18–22 y.o	F	18–23.9 kg/m^2^	Hidden obesity	RCT-parallel	mTRE (ad libitum)	mTRE + EX (11,000–12,499 steps/day	Control	/	/	8-week
11) Kahleova et al. (57)	Czech Republic	*n* = 54	30–70 y.o	F and M	27–50 kg/m^2^	T2D	RCT-crossover	eTRE+ CR (−500 kcal/day)	CR (−500 kcal/day)	/	/	/	24-week
12) Gabel et al. (42)	USA	*n* = 46	25–65 y.o	F	30–45 kg/m^2^	Pre or post menopause	RCT-parallel	mTRE (ad libitum)	Control	/	/	/	12-week
13) Domaszewski et al. (47)	Poland	*n* = 46	65–74 y.o	M	25–29.9 kg/m^2^	Overweight	RCT-parallel	eTRE (ad libitum)	Control	/	/	/	6-week
14) Domaszewski et al. (48)	Poland	*n* = 45	> 60 y.o	F	> 25 kg/m^2^	Overweight	RCT-parallel	mTRE (ad libitum)	Control	/	/	/	6-week
15) Domaszewski et al. (49)	Poland	*n* = 116	65–74 y.o	F and M	25–29.9 kg/m^2^	Healthy/Overweight	RCT-parallel	mTRE (ad libitum)	Control	/	/	/	6-week
16) Ameur et al. (22)	Tunisia	*n* = 64	18–45 y.o	F	> 30 kg/m^2^	Inactive women with obesity	RCT-parallel	eTRE (ad libitum)	eTRE (ad libitum) + HIFT (x3/week, 45–55 min)	/	/	/	12 weeks
17) Lin et al. (44)	USA	*n* = 90	18–65 y.o	F and M	30–50 kg/m^2^	Obese adults	RCT-parallel	mTRE (ad libitum)	CR (−25% kcal)	Control	/	/	6 months
18) Cienfuegos et al. (43)	USA	*n* = 58	18–65 y.o	F and M	30–49.9 kg/m^2^	Obese adults	RCT-parallel	4-h mTRE (ad libitum)	6-ht eTRE (ad libitum)	Control	/	/	8 weeks
19) Tsitsou et al. (16)	Greece	*n* = 59	> 18 y.o	F and M	> 25 kg/m^2^	MASLD	RCT-parallel	eTRE + CR (−500 kcal/d or −20-25%)	mTRE + CR (−500 kcal/d or −20-25%)	CR (−500 kcal/d or −20-25%)	/	/	12-week
20) Črešnovara et al. (20)	Slovenia	*n* = 108	18–60 y.o	F and M	25–35 kg/m^2^	MetS	RCT-parallel	eTRE + CR (−500 kcal/d)	mTRE + CR (−500 kcal/d)	CR (−500 kcal/d)	/	/	3-months
21) Che et al. (39)	China	*n* = 120	18–70 y.o	F and M	> 25 kg/m^2^	Overweight adults, T2D	RCT-parallel	eTRE (ad libitum)	Control	/	/	/	12 weeks
22) Lin et al. (58)	Taiwan	*n* = 63	40–65 y.o	F	> 24 kg/m^2^	Cardiometabolic risk +	RCT-parallel	mTRE + CR (1,400 kcal/d)	CR (1,400 kcal/d)	/	/	/	8-week
23) Irani et al. (51)	Iran	*n* = 56	20–65 y.o	F	26 < BMI < 40	Obese and overweight + Food Addiction	RCT-parallel	mTRE + CR (− 300–500 kcal)	CR (− 300–500 kcal)	/	/	/	8 weeks
24) Cui et al. (24)	China	*n* = 54	20 y.o	F and M	26 kg/m^2^	Overweight and obese young adults	RCT-parallel	eTRE (ad libitum)	eTRE (ad libitum) + RT (x3/week)	Control	/	/	8 weeks
25) Erdem et al. (54)	Turkiye	*n* = 360	18–65 y.o	F and M	27–35 kg/m^2^	Overweight and obese	RCT-parallel	lTRE (ad libitum)	CR	/	/	/	13-week
26) Feehan et al. (55)	Australia	*n* = 32	18–75 y.o	F and M	29.5–29.9 kg/m^2^	NAFLD	RCT-crossover	lTRE (ad libitum)	Control	/	/	/	24-week
27) Jamshed et al. (18)	USA	*n* = 90	25–75 y.o	F and M	30–60 kg/m^2^	Obese adults	RCT-parallel	eTRE + CR (−500 kcal/day)	CR (−500 kcal/day)	/	/	/	14-week
28) Lowe et al. (46)	USA	*n* = 141	18–64 y.o	F and M	27–43 kg/m^2^	Obese adults	RCT-parallel	lTRE (ad libitum)	Control	/	/	/	12 weeks
29) Parr et al. (56)	Australia	*n* = 51	35–65 y.o	F and M	25–45 kg/m^2^	Overweight and Obese, T2D	RCT-parallel	mTRE (ad libitum)	Control	/	/	/	6 months
30) Pavlou et al. (45)	USA	*n* = 75	18–80 y.o	F and M	30–50 kg/m^2^	Obese adults, T2D	RCT-parallel	mTRE (ad libitum)	CR (−25%)	Control	/	/	6 months
31) Celik et al. (53)	Turkiye	*n* = 26	19–32 y.o	F	avg. 26.9 kg/m^2^	Healthy overweight females	RCT-parallel	eTRE (ad libitum)	CR (−500 kcal/day)	/	/	/	8-week
32) Kramer et al. (59)	Canada	*n* = 39	20–70 y.o	F and M	> 25 kg/m^2^	Overweight with early T2D	RCT-crossover	lTRE (ad libitum)	Control	/	/	/	12 weeks
33) Mena-Hernandez et al. (60)	Mexico	*n* = 28	18–50 y.o	F and M	> 25 kg/m^2^	Overweight or obese	RCT-crossover	eTRE (ad libitum)	Control	/	/	/	8 weeks
34) Quist et al. (61)	Demark	*n* = 100	30–70 y.o	F and M	> 25 kg/m^2^	Overweight or obese with high risk of T2D	RCT-parallel	eTRE (ad libitum)	Control	/	/	/	3 months
35) Rizvi et al. (62)	Pakistan	*n* = 90	40–60 y.o	F and M	> 25 kg/m^2^	Overweight and obese	RCT-parallel	lTRE (ad libitum)	CR	Control	/	/	12 weeks
36) Sampieri et al. (63)	Italy	*n* = 41	18–65 y.o	F and M	18 < BMI < 30 kg/m^2^	Healthy adults	RCT-parallel	mTRE (ad libitum)	eTRE (ad libitum)	Control		/	8 weeks
37) Talebi et al. (52)	Iran	*n* = 90	18–40 y.o	F	25–35 kg/m^2^	Females with PCOS	RCT-parallel	eTRE (ad libitum)	eTRE (ad libitum)	CR	/	/	8 weeks
38) Wei et al. (40)	China	*n* = 88	18–75 y.o	F and M	28–45 kg/m^2^	Obese, NAFLD	RCT-parallel	eTRE + CR (1500–1800 kcal/d men and 1,200–1,500 kcal/d women)	CR (1500–1800 kcal/d men and 1,200–1,500 kcal/d women)	/	/	/	6 months
39) Zhou et al. (41)	China	*n* = 74	18–70 y.o	F and M	24.35 kg/m^2^	Stage 1 primary hypertention + without high-risk	RCT-parallel	eTRE (ad libitum)	Control	/	/	/	6 weeks

F = female, M = male, y.o = years old, BMI = body mass index, MetS = metabolic syndrome, NAFLD = nonalcoholic fatty liver disease, T2D = type 2 diabetes, MASLD = metabolic dysfunction associated steatotic liver disease, PCOS = polycystic ovary syndrome, eTRE = early time-restricted eating, mTRE = midday time-restricted eating, lTRE = late time-restricted eating, eTRE + EX = early time-restricted eating + exercise, mTRE + EX = midday time-restricted eating + exercise, RT = resistance training, RCT = randomized controlled trial, HIFT = high intensity functional training, DASH = dietary approaches to stop hypertension.

To determine intervention effects, outcome data were extracted for each intervention arm and comparator arm. Mean change from baseline values and corresponding standard deviations (SDs) were reported or calculated as described in Section 2.7 if not readily available.

### 2.6 Risk of bias assessment

Risk of bias assessment was conducted independently by two authors (MH and WS) and disagreements were resolved by discussion with a third author (YR). Methodological quality was assessed using the Cochrane Risk of Bias 2 (ROB 2). Under ROB 2, each study was assigned judgments of “low risk of bias,” “some concerns,” or “high risk of bias” across five domains: bias arising from the randomization process, bias due to deviations from intended interventions, bias due to missing outcome data, bias in measurement of the outcome, and bias in selection of the reported result. An overall risk-of-bias judgment was also assigned for each study (26).

### 2.7 Certainty of evidence

Grading of Recommendations Assessment, Development, and Evaluation (GRADE) was used to determine the certainty of the body of evidence. The Confidence in Network Meta-Analysis (CINeMA) tool derived from the GRADE approach, was used to assess confidence in the evidence across the network (80). Using CINeMA, six domains were evaluated through qualitative judgments: Within-study bias assesses whether flaws in study design or conduct could result in systematic differences between the observed treatment effect and the true effect, based on the risk-of-bias assessment. Reporting bias evaluates whether the available evidence may be affected by selective publication of studies or selective reporting of outcomes. Indirectness evaluates the transitivity assumption, which considers whether studies comparing different interventions are sufficiently similar in effect modifiers to justify indirect comparisons. Imprecision assesses whether the confidence intervals around treatment effect estimates are sufficiently narrow to support reliable conclusions. Heterogeneity evaluates the extent of variability in treatment effects across studies beyond that expected by chance. Incoherence assesses the agreement between direct and indirect evidence for the same treatment comparison within the network (27). The plausibility of this assumption was supported by the application of strict eligibility criteria, including comparable participant populations, standardized definitions of TRE interventions, and exclusion of populations expected to substantially modify treatment effects. Supplementary characteristics included age, sex, sample size, and duration of study. Imprecision, heterogeneity, and incoherence were subsequently evaluated. Confidence ratings were considered high, moderate, low or very low (28). Minimal important differences (MIDs) for determining clinically important changes in all outcomes were taken from previously published NMAs (Supplementary Table S3) (29, 30).

### 2.8 Data synthesis and analysis

As direct head-to-head comparisons between all interventions were not available, the treatment network was connected through the control group. Network meta-analysis of RCTs was performed using a Bayesian framework in MetaInsight (31). Comparisons included different TRE approaches with exercise versus no exercise, TRE with CR versus no CR, and TRE versus control. The geometry of the treatment network was explored by constructing network plots to visualize the available direct comparisons among interventions. Node size was proportional to the number of participants assigned to each intervention, whereas edge thickness reflected the number of direct comparisons contributing to each treatment contrast.

Random-effects models were used to generate pooled weighted mean differences and 95% credible intervals (CrI) (32). Mean changes from baseline and corresponding SDs were extracted or calculated from each study included. When only the standard error of the mean (SEM) was reported, the SD was calculated using the following formula: SD = SEM × sqrt(*n*), where n represented the number of participants in each study. When the SD of the change from baseline was not reported, it was calculated using:


$${\mathrm{SD}}_{E,\text{change}}=\sqrt{\begin{array}{l} {\mathrm{SD}}_{E,\text{baseline}}^{2}+{\mathrm{SD}}_{E,\text{final}}^{2}-\\ \left(\begin{array}{l} 2\times \text{Corr}\times {\mathrm{SD}}_{E,\text{baseline}}\times \\ {\mathrm{SD}}_{E,\text{final}} \end{array}\right) \end{array}}$$

Where *R* represents the correlation coefficient, which was assumed to be 0.9. This value was estimated from studies that provided sufficient data to calculate the correlation coefficient and was then imputed for the remaining studies with insufficient data. This approach is consistent with guidance from the Cochrane Handbook, which recommends estimating the correlation coefficient from available studies when it is not directly reported (33). If upper and lower limits were reported with the mean, the SD was calculated using the following formula: SD = √*n* × (upper limit—lower limit)/3.92. If the median with upper and lower limits was reported, the estimation was based on the method described by Hozo et al. (34).

Outcomes reported in different measurement units were converted to a common unit using appropriate equations, where possible, to permit pooling using mean differences. When more than one group per category was included in a study, the data were combined as outlined in the Cochrane Handbook (33, 35). Statistical significance was determined using 95% (CrI), and results were considered statistically significant when the 95% CrI did not include the null value.

League tables display the network meta-analysis estimates for all pairwise comparisons. Values are reported as mean differences with corresponding 95% CrI. Each cell compares the intervention in the row with the intervention in the column. Values below zero indicate a greater reduction in the outcome favoring the row intervention, whereas values above zero favor the column intervention. Results were considered statistically significant when the interval did not include zero.

The ranking probabilities of the included arms for BW, BMI, fat %, fat mass, LBM, WC, HC, WHR, FBG, FBI, HOMA-IR, HbA1c, TG, TC, LDL-C, HDL-C, SBP, and DBP were assessed using the surface under the cumulative ranking curve (SUCRA), with higher values indicating a greater probability of being ranked more effective. Local inconsistency was evaluated using the node-splitting approach (36). Subgroup analyses were performed according to baseline energy intake (ad libitum vs. energy prescription) and intervention duration (longer term ≥ 11 weeks vs. shorter term ≤ 10 weeks). Sensitivity analyses were conducted by excluding studies that included participants with normal BMI and studies including healthy participants.

Forest plots for the NMA of all outcomes are provided in the Supplementary Figures S3–S20.

## 3 Results

### 3.1 Screening results of RCTs

**Figure 1:**
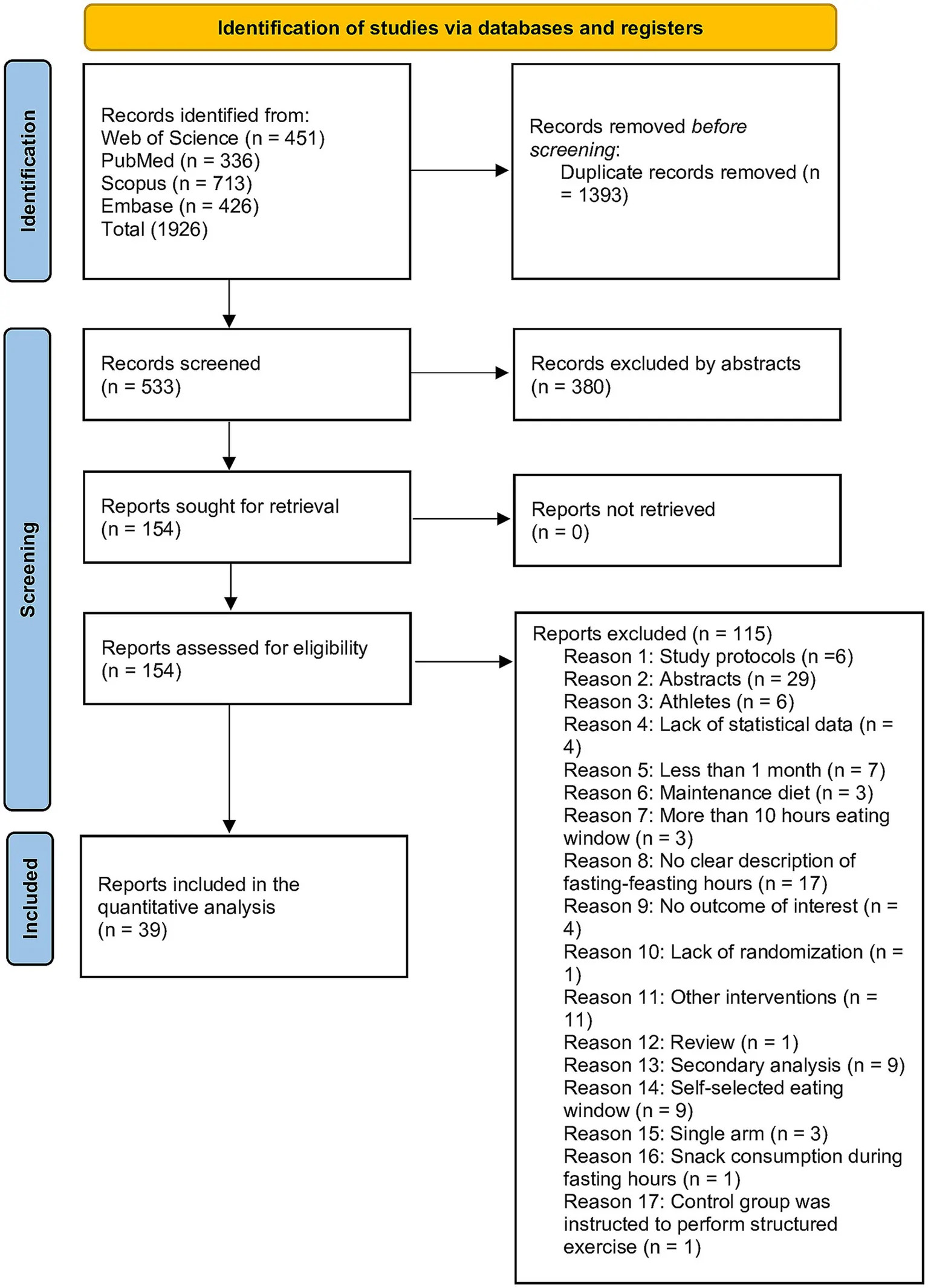
Preferred reporting items for systematic review and meta analysis (PRISMA) diagram.

A total of 1926 articles were identified in the initial search. After removing duplicates (*n* = 1,393), 380 articles were excluded following title and abstract screening. The lTRE + EX node was not included in the network because no published trials evaluating this intervention were available. Following full-text review of 154 articles, 115 were excluded for the reasons detailed in Figure 1. Therefore, 39 articles were included in this NMA.

### 3.2 Studies’ characteristics

Table 2 summarizes the main characteristics of the 39 included studies. Overall, participants’ age ranged from 18 to 75 years. Most studies included both sexes; however, ten studies (25%) included females only and one study (2.5%) included males only. With respect to health status, the included studies enrolled individuals with metabolic syndrome (*N* = 2), T2D (*N* = 5), NAFLD (*N* = 4), polycystic ovary syndrome (*N* = 1), food addiction (*N* = 1), hypertension (*N* = 1), cardiometabolic risk (*N* = 2), and hidden obesity (*N* = 1), with most studies including adults with overweight or obesity. Five studies were crossover RCTs and 34 were parallel-group RCTs. Moreover, twenty-four studies applied eTRE (ad libitum *N* = 16, energy prescription *N* = 8), eighteen applied mTRE (ad libitum *N* = 12, energy prescription *N* = 6), eight applied lTRE (ad libitum *N* = 8), three applied eTRE + EX, two applied mTRE + EX, fourteen applied CR, and twenty-three included control arms. The included studies were conducted in China (11, 12, 14, 21, 24, 37–41), United States (18, 42–46), Poland (47–49), Tunisia (22, 50), Iran (51, 52), Turkey (53, 54), Australia (55, 56), Germany (13), Spain (15), Czech Republic (57), Greece (16), Slovenia (20), Taiwan (58), Canada (59), Mexico (60), Denmark (61), Pakistan (62), Brazil (19), and Italy (63). Finally, trial duration ranged from 4 weeks to 12 months.

### 3.3 Risk of bias assessment

In terms of risk of bias, eleven trials were judged as having some concerns, 24 trials as low risk of bias, and four trials as high risk of bias, as shown in the risk-of-bias graph (Supplementary Figure S1) and risk-of-bias summary (Supplementary Figure S2). Bias due to deviations from intended interventions was frequently judged as high risk because blinding of participants and study personnel was not feasible given the nature of the interventions. Studies that did not adequately describe the randomization process were judged as having some concerns. Final judgments were made according to ROB 2 guidance for each study.

### 3.4 Network diagrams

**Figure 2:**
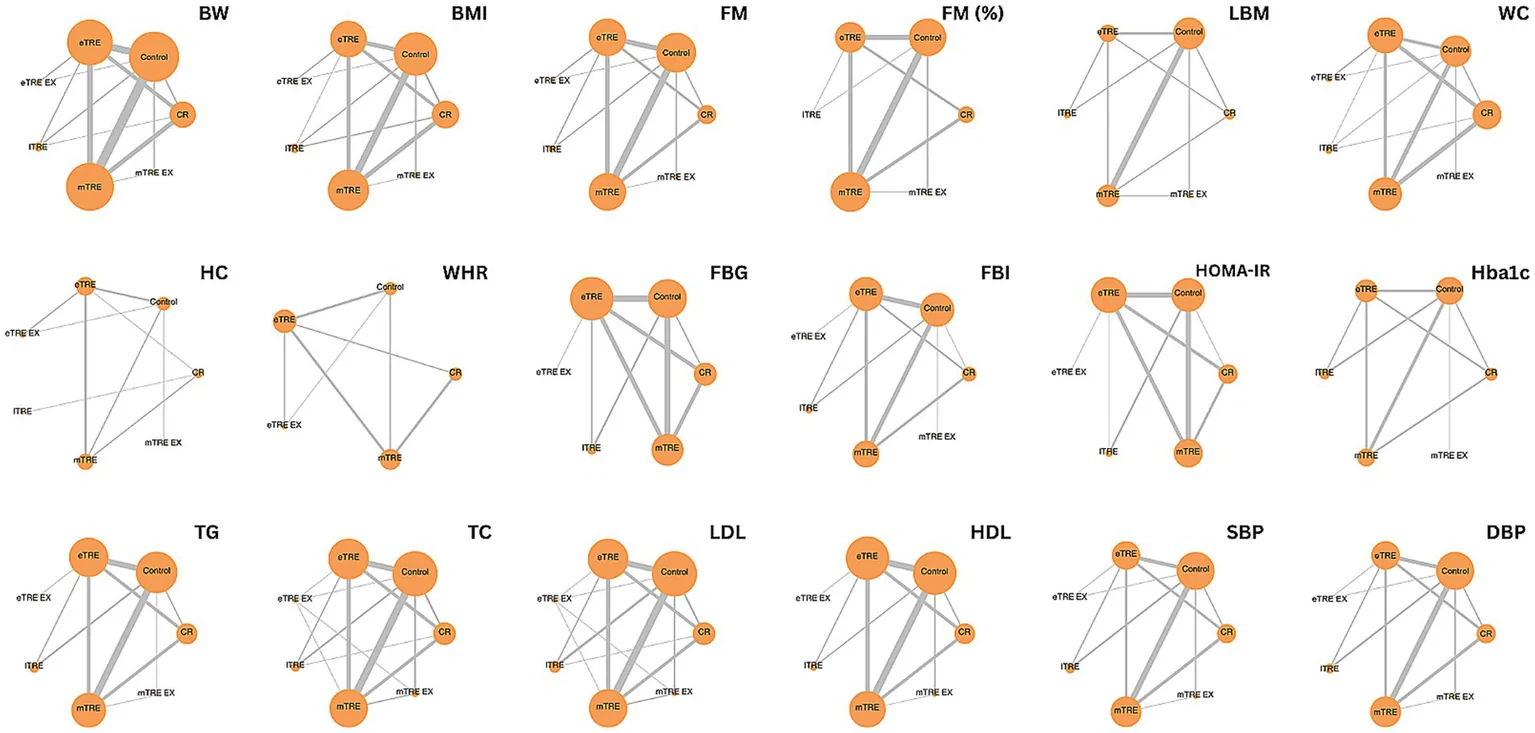
Network plot of direct treatment comparisons for all outcomes. Each node represents an intervention, with node size proportional to the total number of participants randomized to that intervention. The thickness of each connecting line is proportional to the number of direct comparisons between interventions. CR, calorie restriction; eTRE, early time-restricted eating; mTRE, midday time-restricted eating; lTRE, late time-restricted eating; EX, exercise.

The 39 included studies comprised seven interventions (control, CR, eTRE, eTRE + EX, mTRE, mTRE + EX, and lTRE). Figure 2 displays the network of direct comparisons across the interventions. Line thickness is proportional to the number of studies contributing direct comparison evidence, and circle size is proportional to the number of participants randomized to each intervention.

### 3.5 Anthropometric measurements

Data on changes in BW was reported in 35 studies. All interventions aside from mTRE + EX were associated with significantly greater decreases in BW compared with control group (Supplementary Figure S3). eTRE + EX was associated with the greatest decrease (MD = −3.20 kg; 95% CrI: −4.87, −1.59). Rankings from best to worst were eTRE + EX (94%), eTRE (68%), lTRE (55%), mTRE (47%), CR (44%), and mTRE + EX (41%) (Table 3).

**Table 3:** Surface under the cumulative ranking curve (SUCRA) values for each intervention across all outcomes.

Treatment	Body weight	BMI	Fat mass (kg)	Fat (%)	Lean mass	WC	HC	WHR	FBG	FBI	HOMA-IR	HbA1c (%)	TG	TC	LDL-C	HDL-C	SBP	DBP
Control	0.91	0.81	7	23	21	3	47	27	13	8	15	4	35	35	61	32	19	29
CR	44	34	45	62	44	68	54	61	52	32	35	67	55	52	58	24	49	9
eTRE	68	58	49	67	76	67	**71**	**90**	75	57	63	**89**	40	38	56	35	70	60
eTRE + EX	**94**	**84**	**97**	N/A	N/A	**87**	10	35	**93**	**97**	**94**	N/A	**100**	**100**	**100**	**100**	52	**66**
lTRE	55	72	46	72	**78**	41	53	N/A	42	73	69	58	61	57	43	38	41	60
mTRE	47	50	61	**74**	29	33	65	38	24	33	24	33	11	13	16	71	37	63
mTRE + EX	41	51	64	2	51	N/A	N/A	N/A	N/A	N/A	N/A	N/A	47	56	17	50	**83**	65

eTRE = early time-restricted eating, mTRE = midday time-restricted eating, lTRE = late time-restricted eating, eTRE + EX = early time-restricted eating + exercise, mTRE + EX = midday time-restricted eating + exercise, BMI = body mass index, WC = waist circumference, HC = hip circumference, WHR = waist to hip ratio, FBG = fasting blood glucose, FBI = fasting blood insulin, HOMA-IR = homeostatic model assessment for insulin resistance, HbA1c = hemoglobin A1C, TG = triglyceride, TC = total cholesterol, LDL-C = low density lipoprotein cholesterol, HDL-C = high density lipoprotein cholesterol, SBP = systolic blood pressure, DBP = diastolic blood pressure.

Higher SUCRA values indicate a greater probability of being the most effective intervention for a given outcome. Values are presented as percentages, with higher values indicating better rankings. The highest SUCRA value for each outcome is shown in bold. N/A indicates that the intervention was not represented in the corresponding network. The bold values refere to the highest value.

BMI was reported in 31 studies. As with BW, all interventions aside from mTRE + EX resulted in significantly greater decreases in BMI compared with control group (Supplementary Figure S4). eTRE + EX resulted in the greatest decrease (MD = −1.22 kg/m2; 95% CrI: −2.02, −0.43). Rankings from best to worst were eTRE + EX (84%), lTRE (72%), eTRE (58%), mTRE + EX (51%), mTRE (50%), and CR (34%) (Table 3).

Fat mass was reported in 26 studies. All interventions aside from mTRE + EX were associated with significant decreases in fat mass compared with control group. The intervention associated with the greatest decrease was eTRE + EX (MD = −4.65 kg; 95% CrI: −6.19, −3.13) (Supplementary Figure S5). Additionally, eTRE + EX was associated with significantly greater decreases in fat mass when compared with all interventions aside from mTRE + EX. Rankings from best to worst were eTRE + EX (97%), mTRE + EX (64%), mTRE (61%), eTRE (49%), lTRE (46%), and CR (45%) (Table 3).

Fat percentage was reported in 23 studies, none of which utilized an eTRE + EX intervention. Only mTRE and eTRE were associated with significant decreases compared with control (MD = −0.76, 95% CrI: −1.32, −0.27) and (MD = −0.69, 95% CrI: −1.27, −0.15), respectively (Supplementary Figure S6). Rankings from best to worst were mTRE (74%), lTRE (72%), eTRE (67%), CR (62%), control (23%), and mTRE + EX (2%) (Table 3).

Lean body mass was reported in 15 studies which included all interventions except eTRE + EX. Only eTRE was associated with a significant difference in lean body mass compared with control (MD = −0.80 kg; 95% CrI: −1.47, −0.0002) (Supplementary Figure S7). Rankings from best to worst were lTRE (78%), eTRE (76%), mTRE + EX (51%), CR (44%), and mTRE (29%) (Table 3).

WC was reported in 26 studies. All interventions except lTRE were associated with significantly greater decreases in WC compared with control group. The intervention associated with the greatest decrease was eTRE + EX (MD = −4.01 cm; 95% CrI: −7.43, −0.56) (Supplementary Figure S8). Rankings from best to worst were eTRE + EX (87%), eTRE (67%), CR (66%), lTRE (41%), and mTRE (39%) (Table 3).

HC was only assessed by 10 studies. No intervention group was associated with a significant decrease compared with control (Supplementary Figure S9). However, eTRE had the highest SUCRA ranking (71%) (Table 3).

WHR was assessed in 13 studies which did not include lTRE or mTRE + EX interventions. No intervention was observed to have a significant effect compared with control (Supplementary Figure S10). eTRE had the highest SUCRA ranking (90%) (Table 3).

### 3.6 Glucose metabolism

FBG was reported in 28 studies. Compared with control group, significant reductions were observed in eTRE group (MD = −3.64 mg/dL, 95% CrI: −5.93, −1.36) (Supplementary Figure S11). However, SUCRA ranked eTRE + EX as the highest ranking subgroup (93%), followed by eTRE (75%) and CR (52%) (Table 3).

Effects of the interventions on FBI were measured in 21 studies (Supplementary Figure S12). Compared with control group, significant reductions were found with eTRE + EX (MD = −5.43, 95% CrI: −9.41, −1.68; SUCRA 97%), lTRE (MD = −2.49 mIU/L, 95% CrI: −5.13, −0.08; SUCRA 73%), and eTRE (MD = −1.53 mIU/L, 95% CI: −2.99, −0.24; SUCRA 57%) (Table 3).

HOMA-IR was reported in 23 studies and there was no mTRE + EX group for this outcome. Significant reductions compared with control group were found for eTRE + EX (MD = −1.33, 95% CrI: −2.58, −0.08) and eTRE (MD = −0.42, 95% CrI: −0.83, −0.03) (Supplementary Figure S13). SUCRA values ranked eTRE + EX, lTRE, eTRE, CR and mTRE as highest to lowest (94, 69, 63, 35 and 24%, respectively) (Table 3).

Effects of the interventions on HbA1c were reported in 15 studies and there were no eTRE + EX or mTRE + EX groups for this outcome (Supplementary Figure S14). Compared with control group, significant reductions were found only in the eTRE group (MD = −0.37, 95% CrI: −0.62, −0.16; SUCRA 89%) and CR group (MD = −0.29, 95% CrI: −0.61, −0.007; SUCRA 67%) (Table 3).

### 3.7 Lipid metabolism

TC was reported in 30 studies. Compared with the control group, a significant reduction was observed only in the eTRE + EX group (MD = −28.1 mg/dL, 95% CrI: −38.7, −17.4), whereas none of the other groups showed a significant effect on TC (Supplementary Figure S15). SUCRA rankings from highest to lowest were eTRE + EX (100%), lTRE (57%), mTRE + EX (56%), CR (52%), control (35%), and mTRE (13%) (Table 3).

LDL-C was reported in 31 studies. Similar to TC, only the eTRE + EX group showed a significant reduction compared with the control group (MD = −14.9 mg/dL, 95% CrI: −24.2, −5.64) (Supplementary Figure S16). In contrast, mTRE was associated with a significant increase in LDL-C compared with the control group (MD = 3.99 mg/dL, 95% CrI: 0.43, 7.90). None of the other interventions showed significant differences compared with the control group. SUCRA rankings from highest to lowest were eTRE + EX (100%), control (61%), CR (58%), eTRE (56%), lTRE (43%), mTRE + EX (17%), and mTRE (16%) (Table 3).

HDL-C was reported in 29 studies. Compared with the control group, HDL-C was significantly higher in the eTRE + EX group (MD = 19.5 mg/dL, 95% CrI: 12.3, 26.8), whereas no other significant differences were observed among the groups (Supplementary Figure S17). SUCRA rankings from highest to lowest were eTRE + EX (100%), mTRE (71%), mTRE + EX (50%), lTRE (38%), eTRE (35%), control (32%), and CR (24%) (Table 3).

TG was reported in 28 studies. Compared with the control group, a significant reduction was observed in the eTRE + EX group (MD = −0.35 mmoL/L, 95% CrI: −0.65, −0.05) (Supplementary Figure S18). None of the other interventions showed significant differences compared with the control group. SUCRA rankings from highest to lowest were eTRE + EX (100%), lTRE (61%), CR (55%), mTRE + EX (47%), eTRE (40%), control (35%), and mTRE (11%) (Table 3).

### 3.8 Blood pressure

Twenty-three studies assessed the effects of the interventions on SBP. Only eTRE significantly reduced SBP compared with the control group (MD = −2.59 mmHg, 95% CrI: −5.29, −0.005) (Supplementary Figure S19). SUCRA rankings from highest to lowest were mTRE + EX (83%), eTRE (70%), eTRE + EX (52%), CR (49%), lTRE (41%), and mTRE (37%) (Table 3).

A similar number of studies assessed DBP. None of the interventions significantly reduced DBP compared with the control group (Supplementary Figure S20). SUCRA rankings from highest to lowest were eTRE + EX (66%), mTRE + EX (65%), mTRE (63%), eTRE and lTRE (60%), control (29%), and CR (9%) (Table 3).

### 3.9 Inconsistency

Node-splitting results are provided in the Supplementary Figures S21–S36. No statistical evidence of inconsistency was observed using the node-splitting method for FM (kg), LBM (kg), WC, WHR, FBG, FBI, HOMA-IR, HbA1c, HDL-C, TG, SBP, and DBP (Supplementary Figures S23, S25, S26, and S28–S36). However, significant inconsistency was detected for the lTRE vs. control comparison for BW and BMI (Supplementary Figures S21 and S22), the eTRE vs. control, mTRE vs. control, and CR vs. eTRE comparisons for fat (%) (Supplementary Figure S24), and the eTRE + EX vs. control comparison for HC (Supplementary Figure S27). Lastly, the node-splitting method could not be applied to TC and LDL-C because no closed loops were present in the network.

### 3.10 Subgroup and sensitivity analysis

The results of the subgroup and sensitivity analyses, where available, are presented in the Supplementary Tables S4–S45. The *a priori* subgroup analyses by energy prescription showed greater improvements in BW, BMI, fat (%), FM (kg), FBI, and HOMA-IR for eTRE, and in BW, BMI, and fat (%) for mTRE, compared with controls when energy intake was ad libitum. Interestingly, the CR intervention showed numerically more favorable outcomes in the subgroup of studies where TRE was implemented ad libitum than in the subgroup of studies where TRE participants followed a prescribed energy intake. Although the reasons for these differences are not fully clear, factors such as differences in study populations, intervention characteristics, or study design may have contributed to the observed findings.

Subgroup analyses by intervention duration showed greater improvements in most, although not all, outcomes across intervention groups when the intervention duration was shorter, with the exception of eTRE + EX. Shorter interventions may be associated with better dietary adherence, which often declines over time during dietary interventions. Complete results are presented in Supplementary Tables S4–S28.

Complete results of the sensitivity analyses excluding studies that included participants with normal BMI and healthy participants are presented in Supplementary Tables S29–S45. Because only a small number of studies fell into these categories (*N* = 4), their exclusion did not materially affect the primary analysis for most outcomes.

### 3.11 Credibility of the evidence

The certainty of evidence for comparisons among all interventions is presented in the Supplementary Tables S46–S61. For BW, fat %, fat mass, certainty of evidence ranged from very low to high. For BMI, LBM, WC, FBG, FBI, HbA1c, TG, TC, SBP, and DBP, certainty of evidence ranged from very low to moderate. For LDL-C, certainty ranged from very low to high, whereas for HDL-C it ranged from moderate to high. For HOMA-IR, certainty ranged from low to moderate, while for HC it ranged from very low to low. Reasons for downgrading the certainty of evidence from high to moderate, low, or very low included concerns related mainly to within-study bias, indirectness, imprecision, or heterogeneity. For comparisons without direct evidence, certainty was downgraded by two levels because it relied entirely on indirect evidence.

## 4 Discussion

To our knowledge, this is the most comprehensive NMA to summarize the currently available evidence on the effects of various TRE interventions on health outcomes. The analysis included 39 RCTs and 3,236 participants. As discussed above, we also aimed to examine whether meal timing may contribute to outcomes by classifying TRE into three categories: early, midday, and late TRE. Because many recent RCTs have evaluated TRE in combination with exercise interventions, separate nodes were also created for exercise-based TRE intervention to explore any additional benefits of the combined approach.

Across outcomes, eTRE + EX generally ranked among the most favorable interventions, showing improvements in several anthropometric and metabolic outcomes. However, these findings should be interpreted cautiously, as they were based on a limited number of trials and predominantly reflected SUCRA rankings rather than statistically significant differences in direct comparisons. However, the other TRE patterns, whether implemented alone or in combination with EX, as well as CR, showed broadly similar improvements in anthropometric outcomes and lipid profile relative to control. Taken together, these findings suggest that energy restriction may account for a substantial proportion of the overall effects.

Notably, in this NMA, eTRE significantly improved FBG, HbA1c, HOMA-IR, and SBP compared with control, whereas CR and the other TRE variants did not. Although direct comparisons between eTRE and the other active interventions did not reach statistical significance, the direction of effect consistently favored eTRE, which may indicate that meal timing contributes to glucose regulation and insulin sensitivity. These findings are supported by a recent systematic review comparing TRE + CR with CR alone, which reported that insulin sensitivity improved in all studies evaluating eTRE + CR (*N* = 5) relative to baseline (64). It is possible that the glucoregulatory effects of eTRE are mediated, at least in part, by consuming a greater proportion of energy at a time of day that is more closely aligned with circadian rhythms. This may affect hormonal secretion and physiological pathways involving insulin, cortisol, and melatonin, as peripheral tissues possess intrinsic circadian clocks and differ in their metabolic responsiveness across the day (65). Skeletal muscle and liver are examples of peripheral tissues that appear to be more responsive to metabolic stimuli earlier in the day. Accordingly, eTRE may attenuate postprandial glucose and insulin excursions by shifting food intake to earlier hours. In addition, eTRE may reduce oxidative stress and preserve endothelial function, which could translate into improved insulin sensitivity, beta-cell function, and BP (14, 17, 66). Eating earlier may also align food intake with circadian regulation of renal sodium handling, thereby contributing to lower SBP, whereas DBP may be less sensitive to meal timing (67).

If meal timing exerts a strong independent effect on anthropometric measures and lipid metabolism beyond energy restriction, clearer differences between TRE patterns and/or CR would be expected in the current analysis, particularly because most included studies were designed to restrict energy intake, narrow the eating window, or both. Taken together, our findings suggest that meal timing may not play as prominent a role in these outcomes as it does in glucose metabolism. This interpretation is supported by a recent meta-analysis comparing various isocaloric intermittent fasting strategies, including alternate-day fasting and the 5:2 diet, with CR, which found no meaningful differences between interventions (68).

Our subgroup analyses suggested that TRE may be associated with greater weight loss when practiced under ad libitum conditions than when combined with prescribed energy restriction, a finding that is consistent with previous research (69). Similarly, a recent meta-analysis examining changes in caloric intake before and after TRE interventions found that participants tended to reduce their energy intake spontaneously by approximately 400 kcal/day in studies where weight loss was ≥3 kg, whereas smaller reductions were observed in studies where weight loss was <3 kg under ad libitum TRE conditions (70).

These findings do not suggest that TRE combined with prescribed energy restriction is ineffective. Rather, they may point to a psychological or behavioral component of TRE. For example, adhering to a restricted eating window may encourage spontaneous reductions in energy intake without the cognitive burden of calorie counting, which could support long-term adherence (71, 72). Previous research has likewise shown that TRE can induce spontaneous calorie restriction even when participants are allowed to eat ad libitum (73). However, when we performed subgroup analyses stratified by energy prescription across both the intervention and CR groups within the same set of studies, the CR group from studies in which TRE participants followed an ad libitum regimen appeared to perform better than the CR group from studies in which TRE participants were instructed to restrict energy intake (−2.73 vs. − 0.25 kg). This pattern may reflect unmeasured study-level differences or contextual confounding. It is also possible that it arose by chance rather than representing a true effect. Therefore, the impact of ad libitum intake during TRE remains uncertain and requires further investigation.

Consistent with our findings, previous meta-analyses have reported mixed evidence regarding the role of meal timing. In one meta-analysis, eTRE and lTRE did not differ significantly in overweight and obese individuals with respect to BW, FBG, or lipid profile, although eTRE was associated with significant reductions in HOMA-IR and DBP compared with lTRE (74). By contrast, another study reported that eTRE was superior to lTRE for several outcomes, including BW, WC, FM, LBM, FBI, and HbA1c (69, 72). Furthermore, eTRE significantly reduced FBG and HOMA-IR underweight-maintenance conditions in individuals with excess body weight (75). It should also be noted that several of the studies cited above raised the possibility of publication bias based on Egger’s test, although this finding should be interpreted cautiously. More recent studies have shown that eTRE combined with CR significantly improved BW, FM, and WC, but not blood biomarkers, compared with CR alone, supporting the possibility that CR may explain the majority of the metabolic benefits attributed to TRE (76, 77).

Although eTRE is theoretically favored because of its alignment with circadian biology, we did not observe significant differences between eTRE, mTRE, lTRE, and CR for most outcomes. This may indicate that the overall energy deficit overrides any additional benefits attributable to meal timing. In light of the available systematic reviews and meta-analyses, TRE may therefore be neither clearly superior nor inferior to traditional calorie restriction, and the choice between these approaches may be better guided by individual preference, feasibility, and sustainability than by expectations of greater weight-loss efficacy (78). Notably, our findings raise the possibility that exercise may augment the metabolic benefits of eTRE. The mechanisms underlying this effect remain unclear, although both growth hormone and testosterone display circadian rhythmicity. Growth hormone secretion is higher at night, whereas testosterone typically peaks in the morning. Fat oxidation and insulin sensitivity also tend to be greater earlier in the day, which could influence nutrient utilization by skeletal muscle (79). However, these findings should be interpreted cautiously because only three studies included eTRE + EX, and one of these reported BW outcomes only. Moreover, the apparent superiority of eTRE + EX may also reflect residual confounding arising from differences in participant characteristics, exercise protocols, and intervention designs across studies.

### 4.1 Strengths, limitations, and future directions

Strengths of the current NMA include its large and comprehensive synthesis of RCT evidence examining the comparative effects of meal timing strategies, as well as the inclusion of the most up-to-date available studies. Compared with standard pairwise meta-analysis, NMA allows the integration of direct and indirect evidence, improving precision and enabling estimation across all comparisons. Additional strengths include predefined inclusion and exclusion criteria, extensive literature retrieval, and use of *a priori* registered systematic review protocol. We also used an established GRADE-based approach to assess certainty of evidence. Finally, this is the first NMA to distinguish among eTRE, mTRE, and lTRE, which may be important when evaluating the effects of eating time manipulation.

However, several limitations should be acknowledged. Direct evidence was sparse for certain comparisons, particularly those involving lTRE, eTRE + EX, and mTRE + EX, which limited confidence in the estimated rankings and highlights the need for more high-quality RCTs directly comparing these interventions. Consequently, SUCRA rankings, particularly for eTRE + EX, should be interpreted with caution, as they were based on a limited number of trials and a substantial proportion of indirect evidence. In addition, no eligible RCTs evaluating lTRE + EX were identified; therefore, this intervention could not be included in the network, highlighting an important gap in the current evidence base. We were also unable to examine the independent role of fasting duration, as eating windows and dietary intake varied across studies. Both factors may independently influence energy intake, circadian alignment, and metabolic outcomes. Furthermore, because only a small number of exercise-based trials were available, we could not separate effects according to exercise modality, despite evidence that aerobic and resistance training may differentially affect body composition and metabolic markers. The apparently greater effects observed with shorter intervention duration may have been driven by better adherence; however, adherence may not have been consistently assessed or reported across the included studies. Finally, only local inconsistency was explored; future NMAs should assess both local and global inconsistency to strengthen the robustness of the findings.

Taken together, these results suggest that earlier eating patterns may confer additional benefits for glycemic outcomes beyond energy intake alone, supporting a possible role for circadian alignment. However, improvements in metabolic and anthropometric measures appear to be driven predominantly by weight loss regardless of eating window. Due to the limited number of trials, particularly the absence of eligible RCTs evaluating lTRE + EX, further research is needed to clarify how different TRE patterns interact with exercise under controlled energy intake conditions and to better isolate the independent effects of meal timing. Future studies should also account for adherence, as intervention duration and total eating window length may influence compliance and, in turn, affect the observed outcomes.

## 5 Conclusion

This NMA compared several TRE patterns (eTRE, mTRE, and lTRE, with or without EX) with CR and control. Although our analysis did not identify significant differences between TRE patterns and CR, SUCRA rankings consistently favored eTRE, with or without EX, relative to CR, suggesting that meal timing may influence health outcomes. However, certainty of evidence was very low to moderate for most outcomes. In addition, TRE + EX was evaluated in only a small number of RCTs; therefore, these findings should be interpreted cautiously until confirmed by future trials. Future RCTs evaluating these interventions should carefully assess baseline and post-intervention eating windows in both TRE and CR/control groups and minimize unintentional fasting or inadvertent alignment with circadian eating patterns in CR arms (e.g., eating within an 8 a.m.–8 p.m. window). Such design considerations will be important for drawing more definitive conclusions about the additive health effects of meal timing, with or without exercise.

## Data Availability

The original contributions presented in the study are included in the article/Supplementary material, further inquiries can be directed to the corresponding author.
